# Dr. Samuel J. Hayes

**Published:** 1897-08

**Authors:** 


EDITOR AMERICAN JOURNAL OF DENTAL SCIENCE:

### Obituary.

### DR. SAMUEL J. HAYES.

Died at his home in Pittsburg, Pa., June 10, 1897, Dr. Samuel J. Hayes.

Dr. Hayes was born on a large farm near Johnstown, Pa., June 22, 1833. When about eighteen years of age, he entered college, paying his way through a course of study principally by teaching. Subsequently, he finished his training with a theological course and served in the pastorate for several years, being considered successful in his work both in the denomination of United Brethren and in the Baptist. In consequence of a severe bronchial affection, he was compelled to turn from his chosen profession and took up the study of dentistry, which he followed during the remainder of life, about thirty years. The defects of anesthetic agents in general and the crude condition of the science itself, early attracted his notice and he thereafter devoted his professional life to the development of this art. In his numerous writings and lectures before schools and associations, both medical and dental, he advocated and established the bedrock principles of the science, and is considered an eminent authority on the subject, his definitions for anesthesia and asphyxia being so clear and forcible that they are accepted as standard. By his researches and his invention known as "The Hayes Process of Anæsthesia," a means has been given the professional world, of producing a true anesthesia free from peril to operator or patient; this Process is now widely known and used in the United States and, to some extent, in foreigh countries.

Dr. Hayes was editor and proprietor of "The Dental and Surgical Microcosm," a journal depoted to the interests of the dental profession and fearlessly advocating the principles of the art and science of anæsthesia, as they had been opened up and established by him; at the time of his death, he had in process of preparation a book on the subject, which failing health compelled him to defer and which is not yet completed.

